# Successful *en bloc* resection of recurrent hepatocellular carcinoma directly invading the abdominal wall: a case report

**DOI:** 10.1186/1752-1947-9-19

**Published:** 2015-01-22

**Authors:** Aijun Li, Bin Wu, Longjiu Cui, Mengchao Wu

**Affiliations:** Eastern Hepatobiliary Surgery Hospital, The Second Military Medical University, 225 Changhai Road, Shanghai, China

**Keywords:** Hepatectomy, Hepatocellular carcinoma, Recurrence

## Abstract

**Introduction:**

Repeat hepatic resection has previously been reported as the most effective treatment for recurrence of intrahepatic carcinoma. To the best of our knowledge, *en bloc* resection of recurrent hepatocellular carcinoma directly invading the abdominal wall has not been previously reported.

**Case presentation:**

In September 2012, a 64-year-old Chinese male patient was referred to our hospital because of primary hepatocellular carcinoma located in Couinaud’s segments III and V. Our patient first had a hepatectomy of the liver. Ten months later, he presented with an abdominal wall mass and upper abdominal pain. Computed tomography and magnetic resonance imaging scans demonstrated a 10cm tumor in his left liver with extrahepatic metastases in his abdominal wall. It was determined that he had recurrent hepatocellular carcinoma associated with direct invasion into his abdominal wall. He had an *en bloc* left hepatectomy with resection of the tumor in his abdominal wall. A pathological examination of the resected specimen confirmed the diagnosis of hepatocellular carcinoma involving the abdominal wall. Disease-free margins of resection were achieved. Our patient’s postoperative course was uneventful. Eight months after the last surgery, our patient died owing to recurrence and distal metastasis.

**Conclusion:**

Direct invasion of hepatocellular carcinoma into the abdominal wall is rarely encountered. Complete surgical resection should be considered in patients with an appropriate hepatic functional reserve, with consideration of the technical difficulty relating to tumor involvement with surrounding tissues.

## Introduction

Hepatocellular carcinoma (HCC) is one of the most common cancers worldwide, especially in China. Hepatocellular carcinoma directly invading the abdominal wall is rarely reported [1, 2]. To the best of our knowledge, *en bloc* resection of recurrent HCC invading the abdominal wall has never been reported.

## Case presentation

**Figure 1:**
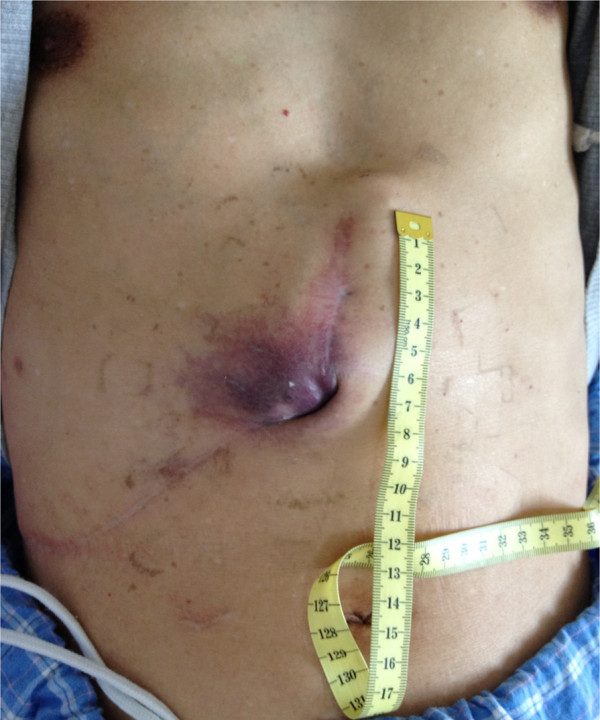
**The original surgical incision with a colored mass in the abdominal wall.**

**Figure 2:**
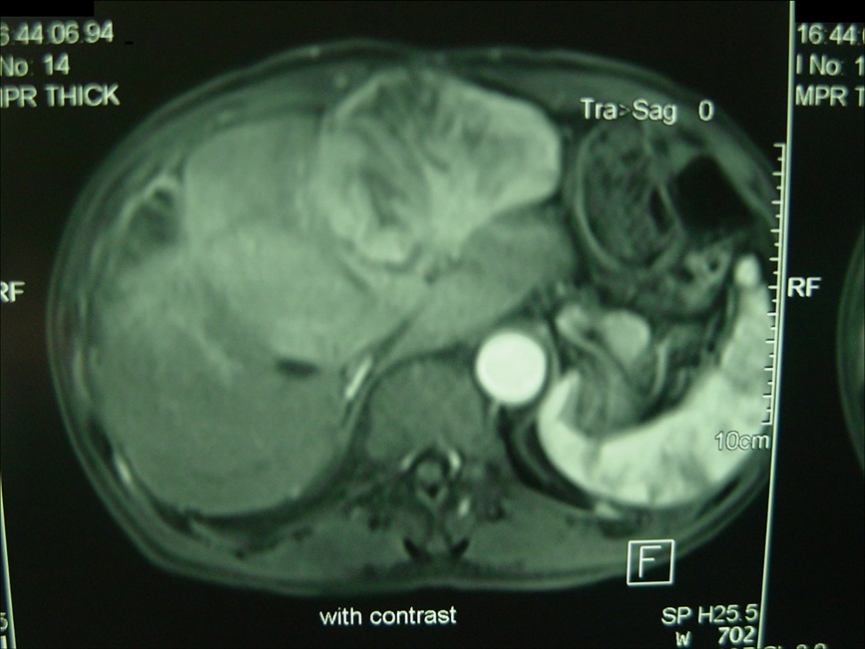
**Magnetic resonance image showing recurrence of the carcinoma in segment IV after 10 months.**

A 64-year-old Han Chinese male patient presented with primary multi-HCC. He had tumors located in Couinaud’s segments III and V, which were treated with local resection. After surgery, he received transcatheter arterial chemoembolization (TACE). After 10 months, he was referred to our hospital in September 2012 with right upper abdominal pain and an abdominal wall mass (Figure 1). He tested negative for the hepatitis B virus but was positive for hepatitis B core antibody. A physical examination revealed the abdominal wall mass to be 7×8cm. Results from routine blood chemistry tests were normal. Levels of tumor markers, such as alpha-fetoprotein, carcinoembryonic antigen and carbohydrate antigen 19–9, were all within the normal range. The functional status of his liver was assessed as class A on the Child-Pugh scale. Abdominal computed tomography and magnetic resonance imaging scans demonstrated a mass with ill-defined borders in the left lobe of his liver and revealed abdominal wall invasion (Figure 2). The maximum diameter of the lesion was 10cm. Because of the extrahepatic tumor invasion and the recurrence of the HCC directly invading his abdominal wall, a hepatectomy and surgical resectioning of his abdominal wall were considered as the procedure of choice.

**Figure 3:**
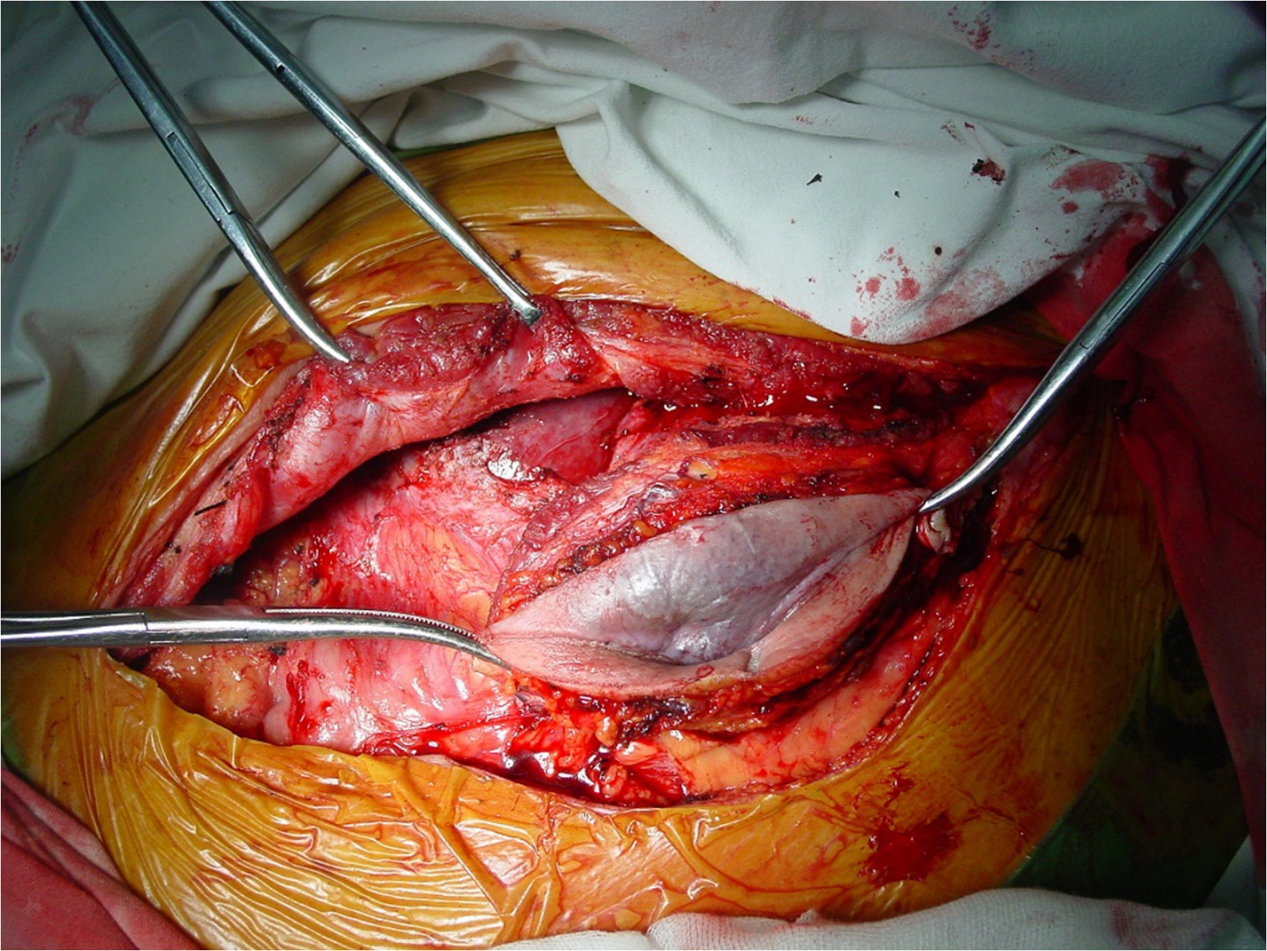
**The incision between the original incision (over a colored mass in the abdominal wall).**

**Figure 4:**
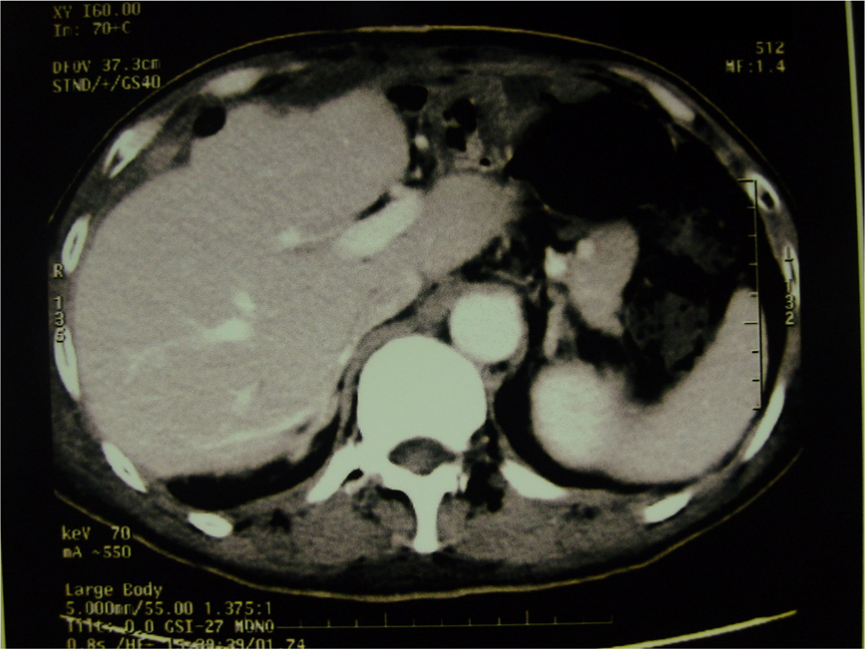
**Imaging showing successful left hepatectomy and resection of the extended skin during the liver operation.** The patient had no recurrence for one month after the second operation.

The second surgery was performed in July 2013. An incision was made between the original incisions, over a colored mass in the abdominal wall (Figure 3). Intra-operative findings included a 10cm tumor involving the left lobe (Couinaud’s segment IV) of his liver. The tumor was invading his abdominal wall. The trunk of his left portal vein, left hepatic artery and left bile duct were divided and ligated. The common venous trunk of his left and middle hepatic veins were then divided and ligated. A liver parenchymal transection was performed without using Pringle’s maneuver. Liver resection was carried out by a clamp-crushing method. Great care was taken to protect his stomach and intestinal wall. Subsequently, a left lobectomy with *en bloc* removal of the abdominal wall was performed. The cut surfaces of his liver were immediately covered by the greater omentum and absorbable hemostatic gauze; because of the large wound (the cut surface), local gauze packing was applied to avoid postoperative bleeding. The operation lasted 2 h. The intra-operative blood loss was 1200mL with six units of red blood cells and four units of fresh plasma transfused during the operation. Continuous abdominal double cannula lavage and low negative pressure drainage were used to drain his abdominal cavity. The abdominal drain and gauze packing were loosened and removed on the third and fifth day after the operation respectively.His postoperative course was uneventful. There were no postoperative complications. Histologic findings were consistent with moderately differentiated HCC invading the abdominal wall. Our patient had a recurrence six months after the second surgery, refused treatment, and died two months later (Figure 4).

## Discussion

Tumor recurrence rates in HCC remain high, at 50% to 60% [3–15], and have even been reported to be over 80% [16]. The main cause of poor survival after liver resection is recurrence. Many authors have suggested that repeat hepatic resection might be the most effective treatment for intrahepatic recurrence, and the five-year survival rate following re-resection ranges from 31% to 69% [17, 18]. Hanazaki *et al*. reported [19] that a curative repeat hepatectomy may be considered the most effective therapeutic modality for recurrent HCC. This was based on the fact that survival was significantly longer in patients with recurrence who had repeat hepatic resection than those who had TACE or percutaneous ethanol injection. Roayaie *et al*. [20] state that a second hepatic resection for recurrent HCC is applicable in about 15% of patients. The procedure is safe and can achieve excellent results in carefully selected patients [20]. Therefore, repeat hepatic resection is accepted as the best treatment for intrahepatic tumor recurrence.

A second surgery is difficult for intrahepatic recurrence and extrahepatic metastasis. Repeat hepatic resection is more difficult than primary resection because of the impaired liver function due to the progression of hepatitis, the presence of adhesions, and modifications in the anatomy caused by the previous operation [21]. In our case, local gauze packing was applied to manage the wound bleeding, which can achieve good outcomes. With removal three to five days after the operation, there is no infection.

In this case we had to carefully consider the design of the incision for the second surgery. Tumors on an incision site will grow quickly and rupture, so surgery is necessary. Sometimes the incision will need a skin flap or graft, particularly if the abdominal wall mass is large and a large defect in the incision is left post-surgery. If a skin flap is not sufficient, reconstruction with further skin replacement for the abdominal wall is required. In our patient, after combined resection of the intrahepatic recurrence and abdominal wall mass, his abdominal tension was reduced. This allowed us to suture the incision so a flap was not required.

## Conclusion

We selected to treat our patient’s extrahepatic tumor invasion of the abdominal wall and HCC recurrence with combined resection. Our experience may be useful in improving the cure and resection rates for liver cancer that is unresectable. We have reported a rare case of a patient who successfully underwent repeat resections for hepatic recurrences directly invading his abdominal wall after hepatectomy for HCC. To prevent postoperative recurrence of HCC, patients require comprehensive treatment.

## Consent

Written informed consent was obtained from the patient’s next of kin for publication of this case report and any accompanying images. A copy of the written consent is available for review by the Editor-in-Chief of this journal.

## Abbreviations

HCC: hepatocellular carcinoma
TACE: transcatheter arterial chemoembolization.
